# BounceBack™ capsules for reduction of DOMS after eccentric exercise: a randomized, double-blind, placebo-controlled, crossover pilot study

**DOI:** 10.1186/1550-2783-6-14

**Published:** 2009-06-05

**Authors:** Jay K Udani, Betsy B Singh, Vijay J Singh, Elizabeth Sandoval

**Affiliations:** 1 Medicus Research LLC, Northridge, California 91325, USA; 2 UCLA School of Medicine, Department of Medicine, Los Angeles, California 90024, USA

## Abstract

**Background:**

Delayed onset muscle soreness (DOMS) is muscle pain and discomfort experienced approximately one to three days after exercise. DOMS is thought to be a result of microscopic muscle fiber tears that occur more commonly after eccentric exercise rather than concentric exercise. This study sought to test the efficacy of a proprietary dietary supplement, BounceBack™, to alleviate the severity of DOMS after standardized eccentric exercise.

**Methods:**

The study was a randomized, double-blind, placebo-controlled, crossover study. Ten healthy community-dwelling untrained subjects, ranging in age from 18–45 years, were enrolled. Mean differences within and between groups were assessed inferentially at each data collection time-point using t-tests for all outcome measures.

**Results:**

In this controlled pilot study, intake of BounceBack™ capsules for 30 days resulted in a significant reduction in standardized measures of pain and tenderness post-eccentric exercise compared to the placebo group. There were trends towards reductions in plasma indicators of inflammation (high sensitivity C-reactive protein) and muscle damage (creatine phosphokinase and myoglobin).

**Conclusion:**

BounceBack™ capsules were able to significantly reduce standardized measures of pain and tenderness at several post-eccentric exercise time points in comparison to placebo. The differences in the serological markers of DOMS, while not statistically significant, appear to support the clinical findings. The product appears to have a good safety profile and further study with a larger sample size is warranted based on the current results.

## Background

Delayed onset muscle soreness (DOMS) is muscle pain and discomfort experienced approximately one to three days after exercise [1]. DOMS is thought to be a result of microscopic muscle fiber tears and is more common after eccentric exercise (the muscle must lengthen or remain the same length against a weight) rather than concentric exercise (the muscle can shorten against a weight load). While DOMS is not a disease or disorder, it can be painful and is a concern for athletes because it can limit further exercise in the days following an initial training [2].

In most cases, DOMS will resolve spontaneously within 3 to 7 days. There is some evidence that ibuprofen, naproxen, and massage may accelerate the resolution of DOMS [2]. Treatment strategies have often integrated multiple therapeutic approaches such as cryotherapy, ultrasound, compression therapy, stretching and deep tissue massage [3-7]. In addition, several dietary supplements have been tested in the treatment of DOMS including protein, vitamin C, proteases (enzymes), phosphatidylserine, chondroitin sulfate, and fish oil, all with variable success [2,8-14]. There is no clear consensus in the extant literature on a method or discipline that can effectively relieve pain following eccentric exercise.

The test product in this study was BounceBack™ capsules; a proprietary dietary supplement combination containing proteolytic enzymes, curcumin, phytosterols from unsaponifiable avocado and soybean oils, vitamin C, and resveratrol.

The current study was designed to induce DOMS in healthy untrained volunteers, assess the level of DOMS through functional and biochemical methods, and determine if the BounceBack™ product is superior to placebo in accelerating recovery from this condition.

## Methods

### Ethics Approval

Ethics approval was obtained through the Copernicus Group IRB in Cary, NC prior to the initiation of the study.

### Study Sample

Ten healthy community-dwelling untrained subjects were enrolled. Subjects were between 18 and 45 years of age (mean 27.73, SD 8.04) and their gender was evenly divided (5 men, 5 women). As the study followed a crossover design, descriptors of the sample are the same, regardless of whether the subject was in the placebo arm or the active arm of the study.

### Investigational Products

BounceBack™ is a dietary supplement sold in capsule form by Mannatech, Incorporated (Coppell, TX). The two capsule daily serving contained 258 mg of a proteolytic enzyme blend that includes bromelain as well as proteases from *Aspergillus melleus *and *A. oryzae*. The ingredients of the two capsules also included 421 mg of tumeric extract (root/rhizome; standardized to 95% curcumoids), 90 mg of a phytosterol blend (beta-sitosterol, campesterol and stigmasterol), 20 mg vitamin C and 6 mg Japanese knotweed extract (root; standardized to 20% resveratrol). The placebo, which was encapsulated maltodextrin, looked identical to the test product.

### Study Design

The study was a randomized, double-blind, placebo-controlled, crossover study. Mean differences within- and between-groups were assessed inferentially at each data collection time-point using t-tests for all outcome measures. Given the small number of subjects in this pilot study, the use of an ANOVA or ANCOVA to run repeated measures was deemed inappropriate.

During the screening visit (Visit 0) subjects were assessed for eligibility, given a physical exam, randomized into the test or placebo group, and given the appropriate investigational product. Subjects received an electronic SenseWear™ armband (BodyMedia, Pittsburgh, PA) to record activity data. In order to limit the variable impact of diet on plasma markers of inflammation, subjects were given an identical set of frozen foods to consume for each of the 24-hours periods prior to their day 30 exercise visits.

During each arm of this crossover study, subjects took the investigational study product for 30 days before returning for their exercise visit (day 30). After the exercise visit, they returned on days 31, 32 and 33 for additional assessments and blood draws. After completing the day 33 visit, subjects underwent a two week washout of the study product before beginning the second arm of the study, which followed the identical timetable.

The eccentric exercise protocol consisted of repeated quadriceps squats using a Smith Machine: a barbell fixed within steel rails, so that it can only move vertically. At the screening visit subjects were asked to complete the maximum number of squats that they could perform in a five minute period. This effort was regarded as submaximal and therefore at the two subsequent exercise visits, subjects were required to perform twice as many squats as they had performed during the screening visit.

### Outcome Measures

The primary outcome measures were assessments of pain and tenderness. Pain was assessed using a Visual Analog Scale (VAS) pain score comprised of four subscales (current pain, least amount of pain, most amount of pain, and whether pain was interfering with function) each of which was measured on a scale from 0 (no pain) to 10 (worst possible pain). Tenderness was assessed using an algometer (set at level 4) to experimentally induce pain on a predefined point on the patellar tendon five centimeters above the center of the patella. Subjects then ranked their pain perception on a scale from 0 to 10. On day 30, assessments were taken at baseline (pre-exercise), and again at six hours post-exercise. Subjects returned for further assessments 24, 48 and 72 hours post-exercise for of each arm of the study. Secondary outcomes included assessments of inflammation, muscle damage, flexibility, and the amount of energy expended prior to exercise.

Blood was drawn on day 30 (pre-exercise), and 6, 24, 48 and 72 hours post exercise. Assays were performed for creatine phosphokinase (CPK), myoglobin, high sensitivity C-reactive protein (hs-CRP), tumor necrosis factor (TNF)-alpha, interleukin (IL)-1, and IL-6. Flexibility was measured using standard flexion and extension measures and range of motion (ROM) assessments for both legs. Data on energy expenditure (EE) was collected using the SenseWear ™ armband device. This armband, which has been validated by several studies [15-17], uses a 2-axis accelerometer, a heat flux sensor, a galvanic skin response sensor, a skin temperature sensor and a near-body ambient temperature sensor to capture data. These data, in combination with body weight, height, handedness and smoking status, are used to calculate EE. The armband was placed on the upper arm and worn continuously for the 48 hours prior to exercise in order to assess whether the level of activity prior to exercise impacted any of the primary or secondary outcomes.

To determine the safety profile of the product compared with placebo, the following assays were performed on blood drawn at baseline and again at 72 hours post-exercise in each arm of the study: complete blood count (CBC), kidney function, liver function, prothrombin time/partial thromboplastin time (PT/PTT), and urinalysis. Adverse Event monitoring was conducted throughout the study using standardized assessments at each visit.

## Results

### Safety Assessment

No adverse events were reported during the study period. In addition, no clinically significant changes were seen in any of the laboratory safety values (CBC, liver function, kidney function, PT/PTT, and urinalysis) in either group.

### Pain Assessment

Four different VAS questions were asked regarding pain perception and a total score was calculated by adding the scores from the four individual pain questions. The first three VAS scores were concerned with current pain, least pain, and worst pain experienced by the subjects. The fourth question addressed the degree to which pain was interfering with function. These four questions were asked at each outcome collection time period. All four scores were combined to create the VAS sum score.

Subjects taking the test product experienced significant reductions in overall current pain at six hours (p = 0.039) and 48 hours (p = 0.001) post exercise. Overall current pain was not significantly different at any other measurement point. Subjects' taking the test product reported least pain scores significantly lower than placebo at 6 hours (p = 0.002) and 48 hours post exercise (p = 0.004). All other comparison points were not significantly different between groups for least pain. There was no significant difference in response to the worst pain question between groups at any measurement period. The test product group reported significantly less impact of pain on function at the pre-exercise evaluation (p = 0.005) and at the 6 hour post-exercise time point (p = 0.047). Differences between groups were non-significant at all other time points.

**Figure 1:**
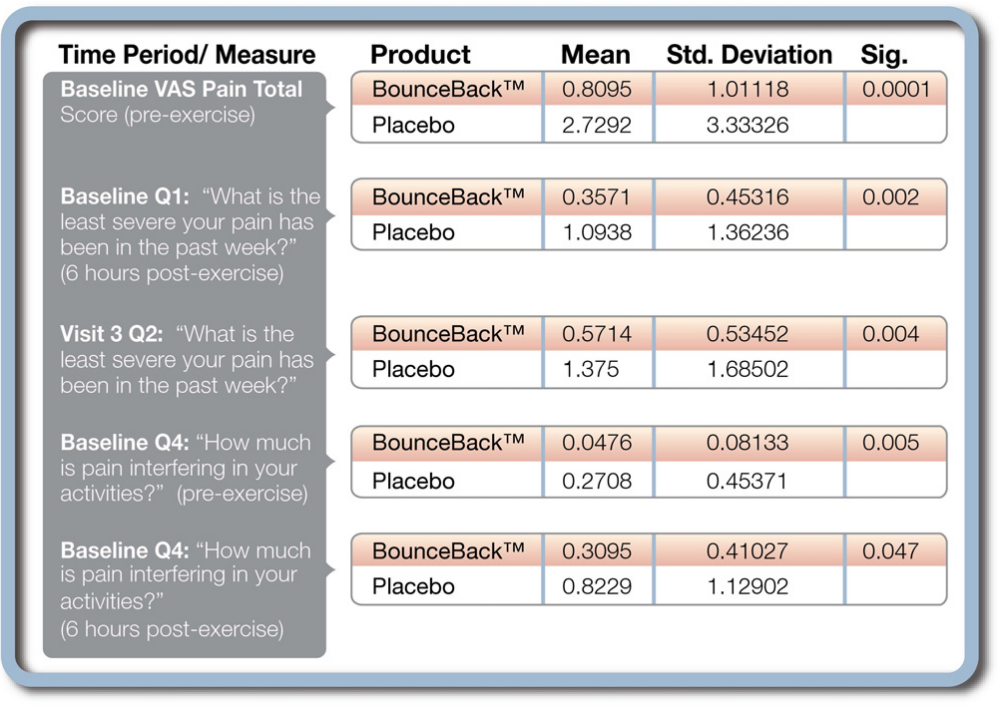
**Pain assessment – visual analogue scale results**.

When the VAS scores were summed, the pre-exercise and 48 hours post-exercise totals were significantly lower in the test product group (p = 0.0001 and p = 0.05, respectively). All other differences for the VAS sum were non-significant. (Figure 1)

### Tenderness Assessment

The study product group demonstrated significantly less tenderness at 24 hours after exercise (p = 0.042). No differences between groups were seen at any other time point.

### Inflammation Assessment

**Figure 2:**
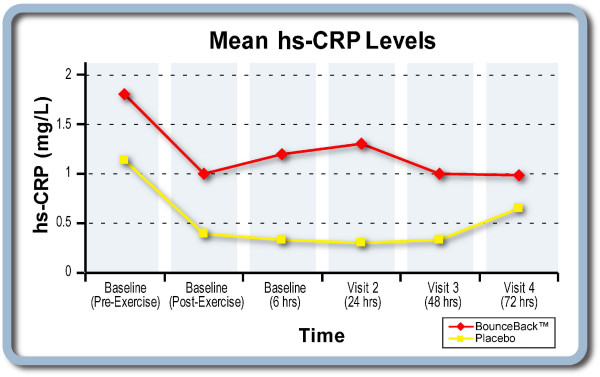
**Mean hs-CRP levels**.

There were no statistically significant differences between groups for plasma markers of inflammation (hs-CRP, TNF-alpha, IL-1, IL-6). The placebo group sustained a small post-exercise elevation in hs-CRP through the 72 hour visit, while the test product group demonstrated a trend toward a reduction in hs-CRP during the same time period (Figure 2).

### Muscle Damage Assessments

**Figure 3:**
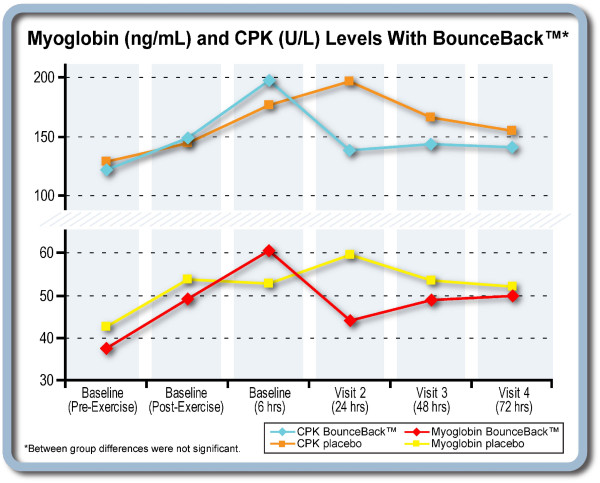
**Myoglobin and CPK**.

Plasma CPK values were not significantly different between the test product and placebo groups from the pre-exercise period to 72 hours post exercise. However, Figure 2 demonstrates that by 24 hours post-exercise, the placebo group trended toward a higher level of CPK than the BounceBack™ group. This trend continued through the 72 hour assessment. A similar phenomenon was observed for plasma myoglobin, which trended higher at 24 and 72 hours post-exercise in the placebo group. While not significant, these trends both suggest sustained increased muscle damage in the placebo group (Figure 3).

### Flexion and Extension Measurements

The pre-exercise flexion measurements were equal between groups for the left leg, but were significantly greater for the placebo group on the right leg (p = 0.049). Post-exercise, all flexion measurements were not significantly different, with the exception of the 6-hour right leg flexion measurement which was significantly greater in the test product group (p = 0.045). When calculating the difference between pre-exercise and all post-exercise time point flexion measurements, all values were not significantly different between groups with the exception of the 6 hour post-exercise right leg flexion measurement which was significantly (p = 0.004) in favor of the test product.

### Energy Expenditure Data

**Figure 4:**
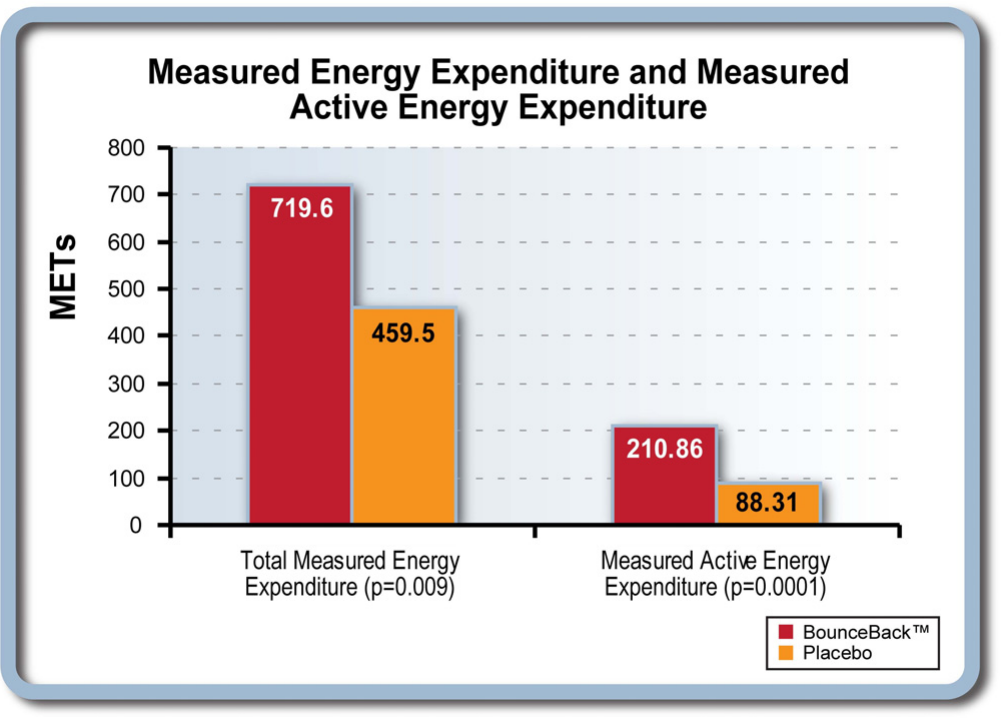
**Energy expenditure 48 hours before exercise protocol**.

Data analysis from the SenseWear™ Armband revealed that there was no significant difference in Total Energy Expenditure (EE) between the two groups in the 48 hour period prior to exercise. EE was composed of Measured Energy Expenditure plus Offbody Energy Expenditure. The BounceBack™ group demonstrated a greater Measured Energy Expenditure compared to the placebo group: METs (physical activity duration and levels) of 720 ± 1012 (mean ± standard deviation) compared to 460 ± 785 (p = 0.009) (Figure 4). In contrast, the Offbody Energy Expenditure was greater for the placebo group: 661 ± 800 compared to 493 ± 637 (p = 0.009). The BounceBack™ group demonstrated greater Active Energy Expenditure: METs 211 ± 322 compared to 88 ± 173 for the placebo group (p = 0.009) (Figure 3). The Average METs was greater for the BounceBack™ group compared to the placebo group: 1.9 ± 1.5 compared to 1.3 ± 1.0 (p = 0.013).

## Discussion

In this small pilot study, when compared with placebo, the BounceBack™ product groups experienced significant reductions in standardized measures of pain and tenderness following eccentric exercise. The differences in the serological markers of DOMS, while not statistically significant, appear to support the clinical findings. There were no observed side effects.

BounceBack™ capsules contain proteolytic enzymes, curcumin, phytosterols from unsaponifiable avocado and soybean oils, vitamin C, and resveratrol: ingredients intended to provide benefit to individuals pursuing an active lifestyle. Two previous short-term clinical studies have examined the effects of ingestion of larger amounts of proteolytic enzymes on DOMS. A placebo-controlled study examined the effects of four days of protease supplementation on muscle soreness and contractile performance after downhill running [11]. One day before exercise and for three days after exercise, ten male subjects consumed two enzyme tablets (325 mg pancreatic enzymes, 75 mg trypsin, 50 mg papain, 50 mg bromelain, 10 mg amylase, 10 mg lipase, 10 mg lysozyme, 2 mg chymotrypisn) (providing a total of 2.144 g/day proteases, 40 mg/day amylase and 40 mg/day lipase) or a placebo four times a day. The treatment group had superior recovery of contractile function and lower subjective pain ratings compared to the placebo group.

Another study examined the potential effects of ingestion of a protease supplement by men for three days on DOMS created by maximal eccentric isokinetic action of the forearm flexor [10]. The supplement provided 342 mg of Protease 6.0 and 340 mg Protease 4.5: a total of 682 mg/day proteases derived from fermentation of *Aspergillus oryzae*. In this double-blinded, placebo-controlled, crossover design study, isometric forearm flexion strength was greater for the supplement group than for the placebo group. There was no effect on subjective pain ratings or blood markers of muscle damage (plasma creatine kinase activity or myoglobin concentrations).

In addition to protease enzymes, BounceBack™ capsules contain curcumin. Curcumin has been reported to reduce pain and swelling associated with inflammation [18]. In a mouse model, curcumin reduced inflammation and performance deficits in mice that performed eccentric exercise (downhill running) [19]. Other ingredients in BounceBack™, namely vitamin C and resveratrol, offer antioxidant support. Antioxidants offer resistance to free radical proliferation, a theoretical cause of tissue damage [2]. In addition, BounceBack™ capsules contain phytosterols from unsaponifiable avocado and soybean oils (ASU). ASU has demonstrated anti-inflammatory activity [20] and effectiveness in the treatment of osteoarthritis [21].

In the present study BounceBack™ capsules demonstrated significant improvement in subjective pain and tenderness, with no significant improvement in levels of markers of inflammation, muscle damage or muscle flexion. BounceBack™ contains a multiple ingredients that are indicated for relieving the symptoms of DOMS. The results of this study adds to previous clinical studies conducted on the use of protease supplements for symptoms of DOM. Compared with those studies [10,11], these effects were achieved for both men and women following longer term intake of a smaller amount of protease enzymes along with supplemental ingredients.

One drawback of this study was the small sample size. Though the differences in the serological markers for inflammation and muscle damage were not statistically significant, a larger sample size may have produced results in favour of the active product. A subsequent study with a larger sample based on power calculations from this study may offer a better idea of the scope of the effectiveness of BounceBack™ product to mediate muscle damage following eccentric exercise.

## Conclusion

The BounceBack™ product was able to significantly reduce standardized measures of pain and tenderness at several post-eccentric exercise time points, compared to placebo. The differences in the serological markers of DOMS, while not statistically significant, appear to support the clinical findings. The product appears to have a good safety profile and further study with a larger sample size is warranted based on the current results.

## Competing interests

No competing interests are declared for JKU, BBS, VJS and ES.

## Authors' contributions

JKU conceived of the study, and participated in its design and coordination and helped to draft the manuscript. BBS participated in the design of the study, performed the statistical analysis, and drafted the manuscript. VJS participated in the statistical analysis and in the drafting of the manuscript. ES participated in the coordination of the study.
